# Control of CREB expression in tumors: from molecular mechanisms and signal transduction pathways to therapeutic target

**DOI:** 10.18632/oncotarget.7721

**Published:** 2016-02-25

**Authors:** André Steven, Barbara Seliger

**Affiliations:** ^1^ Institute of Medical Immunology, Martin Luther University Halle-Wittenberg, Halle (Saale), Germany

**Keywords:** transcription factor, CREB, tumor, regulation, target, prognostic marker

## Abstract

The cyclic AMP response element binding (CREB) protein has pleiotropic activities in physiologic processes. Due to its central position downstream of many growth signaling pathways CREB has the ability to influence cell survival, growth and differentiation of normal, but also of tumor cells suggesting an oncogenic potential of CREB. Indeed, increased CREB expression and activation is associated with tumor progression, chemotherapy resistance and reduced patients' survival. We summarize here the different cellular functions of CREB in tumors of distinct histology as well as its use as potential prognostic marker. In addition, the underlying molecular mechanisms to achieve constitutive activation of CREB including structural alterations, such as gene amplification and chromosomal translocation, and deregulation, which could occur at the transcriptional, post-transcriptional and post-translational level, will be described. Since downregulation of CREB by different strategies resulted in inhibition of cell proliferation, invasion and induction of apoptosis, the role of CREB as a promising target for cancer therapy will be also discussed.

## INTRODUCTION

The 43 kD cyclic AMP (cAMP)-responsive element binding protein (CREB), first identified 1987, belong to the large family of basic leucine zipper (bZIP)-containing transcription factors (TF) including c-jun, c-fos and c-myc. It is a crucial transcription factor, which regulates a wide range of biological processes to orchestrate proper cell differentiation and cell growth. Genome wide analyses of the CREB-binding sites identified more than 4.000 genes with cAMP-responsive elements (CRE) in their promoters suggesting that CREB controls not only the regulation of immediate early genes as primarily expected [1, 2].

CREB is currently viewed as a multifaceted protein that associates with diverse proteins to direct biological distinct activities in a context-dependent manner. Its importance for basic cellular function and homeostasis is further strengthened by the lethality of CREB knock out mice [3]. In addition, its overexpression is associated with an increased cell proliferation, reduced apoptosis and enhanced migration. Thus, there exists evidence for a causal link between CREB activation, tumor initiation and progression. This review focusses on (i) how CREB expression is controlled and (ii) how protein-protein interactions dynamically regulated in a spatiotemporal manner have endowed the CREB protein with a plethora of functions with particular emphasis on the tumor promoting properties of CREB. In addition, its use as a prognostic biomarker and therapeutic target of tumors is discussed.

## ESSENTIAL FEATURES AND FUNCTIONS OF CREB

CREB is a modular protein consisting of a kinase-inducible domain (KID), two glutamine-rich domains and a bZIP domain. The KID- and glutamine-rich domains are essential for the transactivation and phosphorylation of CREB. The transcriptional activity of CREB is induced upon a reversible phosphorylation at various serine residues, in particular at serine 133 and serine 142, by various kinases, such as protein kinase A (PKA), protein kinase B (PKB/AKT), the mitogen-activated kinase (MAPK) and the 90 kD ribosomal S6 kinase [4–10]. Phosphorylated CREB (pCREB) interacts with diverse transcriptional co-activators including the histone acetyltransferase CREB-binding protein CBP/p300 via the kinase-inducible domain (KID) in CREB and the KID-interacting domain (KIX) in CBP [11–13] thereby subsequently increasing its transcriptional activity. The CREB/CBP complex recruits the transcriptional machinery at the CRE site of gene promoters for the initiation of the CREB-dependent gene transcription [2].

Since CREB is a general transcriptional activator involved in the modulation of the histone H3 and H4 methylation leading to the initiation and maintenance of the chromatin recruitment to the transcriptional apparatus [13], it could regulate a large number of physiological processes dependent on its cellular localization and time-dependent phosphorylation pattern. These include cell proliferation, cell cycle, metabolism, DNA repair, differentiation, inflammation, angiogenesis, immune responses and survival.

## ROLE OF CREB IN THE TUMOR DEVELOPMENT

**Figure 1:**
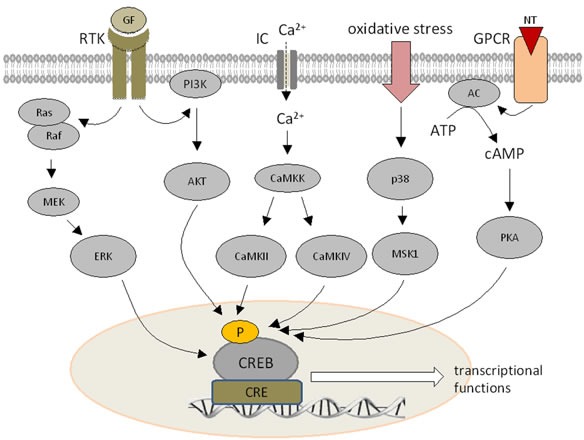
Signal transduction pathways modulating CREB expression Growth factors (GF) can bind to a membrane-bound receptor, which activates the PI3K-AKT or Ras-MEK-ERK pathways. Ca^2+^ influx increases the activity of calcium dependent kinases. Hormone receptors and G protein coupled receptors stimulate cAMP synthesis by adenylate cyclase leading to the activation of PKA. All signal transduction pathways can phosphorylate CREB at different serine residues.

Next to its physiological role CREB is also involved in the malignant transformation of cells, since its frequent and persistent activation is sufficient to convert normal cells into tumor cells. This is mediated by an aberrant activation of components of the cAMP signal transduction relevant pathways, such as G-coupled, receptor tyrosine kinase (RTK) and cytokine/JAK/STAT signaling pathways, but also downstream signaling pathways (Figure 1).

CREB overexpression was found in many solid tumor types like non-small lung carcinoma (NSCLC), glioblastoma, mammary carcinoma, melanoma and diffuse malignant mesothelioma when compared to adjacent normal tissues [8, 10, 14–22] as well as in hematopoietic malignancies [23–26]. This was accompanied by enhanced cell proliferation, reduced sensitivity to undergo apoptosis, increased angiogenesis and radiation-induced differentiation [27]. Furthermore, CREB overexpression is associated with clinicopathological parameters including tumor stage, grade, metastasis, enhanced development of recurrences, a worse prognosis and a reduced survival of tumor patients [17, 28–31]. This was due to a CREB overexpression-mediated upregulation of downstream target genes of CREB carrying CRE elements in their promoters. Chromatin immunoprecipitation (ChIP) and a combination of ChIP with SAGE identified a large number of CREB targets involved in the neoplastic phenotype, clonogenic potential, apoptosis resistance, and abnormal growth properties [32–35]. Furthermore, CREB overexpressing transgenic mice developed myeloproliferative disorders [23].

In addition, CREB has been shown to play a key role in the development of resistances against inhibitors of the Raf-MEK-ERK and PI3K/AKT signal pathways [36, 37]. The resistance against MAPK inhibitors could be enhanced by CREB in mammary carcinoma, which was moreover associated with an altered histone acetylation [36, 38]. Furthermore, downregulation of CREB caused an altered expression of BRAC1 and an increased expression of aromatase, a key enzyme of the estrogen biosynthesis, which is transcriptionally regulated by CREB and associated with the development of resistances to tamoxifen treatment [37].

## MOLECULAR BASIS OF CREB REGULATION

**Figure 2:**
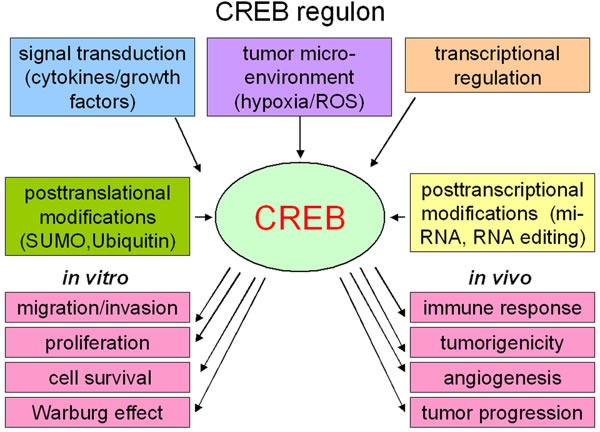
Regulation of CREB (The CREB regulon) Cytokines and growth factors as well as the tumor microenvironment can influence the CREB activity by different signal transduction pathways. Post-transcriptional alterations, like micro-RNAs or RNA-editing, can influence the CREB protein expression, while post-translational modifications regulate the stability or degradation of CREB. In *in vitro* cell culture CREB increases the migration and invasion potential of the cells and is important for the cell proliferation and cell survival. CREB activity can further induce the Warburg effect. Additionally, *in vivo* the transcription factor CREB is linked with the immune responses, tumorigenicity as well as angiogenesis and therefore with tumor progression.

The expression and activity of CREB is dynamically regulated by diverse mechanisms (Figure 2). CREB responds to cAMP, intracellular Ca2^+^, various growth factors, such as nerve growth factor (NGF), fibroblast growth factor (FGF) and the insulin growth factor 1 as well as cytokines, like IL-4, IL-10, IL-13 and transforming growth factor (TGF)-β thereby activating gene transcription [39–41]. The FGF- and stress-mediated regulation of CREB occurs via the MAPK kinase pathway [9, 42].

Both *in vitro* and *in vivo* studies of tumors and corresponding non-malignant tissues as well as of tumor cell lines demonstrated high levels of CREB expression. The underlying molecular mechanisms of CREB overexpression in tumors have not yet been identified in detail. In contrast to the CREB-binding protein (CBP), which is often mutated in tumors [43, 44] amplifications and/or deletions in CREB have only been rarely detected [21] suggesting that deregulation processes might be the major cause of increased expression and function of CREB in tumors. Although in most tumors a concordant upregulated CREB mRNA and protein expression was found, this linear correlation was not always detected in tumors suggesting additional regulatory mechanisms affecting protein levels [45]. Thus next to the transcriptional regulation, the expression of CREB could be also controlled at the post-transcriptional level.

### Post-transcriptional regulation of CREB by microRNAs

MicroRNAs (miRs) representing small non-coding RNA molecules interact with the 3′ untranslated region (UTR) of their target mRNAs and are involved in the regulation of > 50 % of all genes. Thus, miRs might control many cellular and pathophysiologic processes including the initiation and progression of tumors. During the last years miRs have been identified, which are deregulated by CREB or have CREB as direct target due to binding to its regulatory sequences at the 3′-UTR (Table 1). Using *in silico* prediction by different algorithms CREB expression could be regulated by different miRs known to be frequently downregulated in tumors, such as miR-181b, miR-128, miR-124, miR-34b, miR-23a, miR-200b, miR-203 and miR-301 [21, 46–49]. In some studies luciferase reporter assays confirmed the interaction of these miRs with the 3′-UTR of CREB. Overexpression of these miRs significantly modulated the expression of CREB, which was associated with altered growth properties of tumor cells thereby suggesting that the miR-mediated deregulation of CREB contributes to tumorigenesis. For example miR-200b, miR-301 and miR-343 have tumor suppressive activity by targeting CREB. Overexpression of these miRs caused an inhibition of tumor cell growth and/or soft agar colony formation *in vitro* and a reduced tumorigenesis *in vivo* [49]. This could be associated with suppressed expression levels of CREB target proteins and their related pathways [47]. Furthermore, the inverse expression of CREB and miR-200b had also a prognostic value in astrocytoma [49].

**Table 1:** Identification of CREB-regulating miRs in human tumors

Name	Cell line/Tumor	Reference
miRNA-181b	adenocarcinoma	[104]
miRNA-34b	AML	[20]
miRNA-181a	PC12 (pheochromocytoma)	[57]
miRNA-9	glioblastoma	[21]
miRNA-200b	glioblastoma	[45]
miRNA-372	hepatocellular carcinoma	[105]

CREB could also regulate the expression of miRs, such as miR-9, which modulates different physiologic and pathophysiologic processes including the differentiation and function of myeloid-derived suppressor cells (MDSC) [50]. Loss of miR-9 suppresses proliferation, promotes migration of progenitor cells *in vitro* [51] and coordinates the proliferation of glioma cells [21]. In pancreatic cancer, the CREB-dependent induction of miR-373 promotes pancreatic tumor growth *in vitro* and *in vivo* [52]. In melanoma cells CREB has been shown to suppress the expression of the RNA-editing enzyme ADAR1 *in vitro* and *in situ*, while the restoration of its expression reduced melanoma growth and metastasis formation *in vivo*. This was accompanied by RNA-editing of miR-455-5p, which occurs in less aggressive, but not in strong aggressive metastatic melanoma [53]. In addition, the RNA-binding protein tristetraprolin (TTP), a tumor suppressor gene, regulates CREB activity suggesting that low TTP levels represent a potential biomarker for human cancers with poor outcome [54].

### Post-translational modifications

Recently it has been described that CREB expression could be regulated by miRs [20, 21, 55–57]. Next to its post-transcriptional control, CREB expression can be also post-translationally regulated by different extracellular signals, in particular by factors of the tumor microenvironment, like hypoxia, pH and oxidative stress [58]. So far, the best analyzed post-translational modifications (PTMs) of CREB are phosphorylation and ubiquitination, which have been also shown to be altered in tumor cells.

In addition, other PTMs, like methylation, glycosylation and SUMOylation, have also been shown to influence the activity of CREB [55, 59–67]. This might have functional consequences due an altered CREB-regulated gene transcription, including that of nuclear and mitochondrial genes, which is accompanied by changes of protein degradation as well as protein/protein interactions. However up to now, a direct link to tumor initiation and progression has not yet been found.

#### Phosphorylation

CREB is a substrate of various kinases. Although the phosphorylation of CREB can occur at different serine residues, its phosphorylation at Ser^133^ and its functional consequences have been investigated in great detail. In particular, CREB Ser^133^ has been often shown to be overexpressed in human tumors. This modification might result in conformational changes, which might be associated with its functional activity, localization and/or stability. In addition, there exist a number of other serine residues in the KID domain, which could be also phosphorylated. However, neither their expression pattern nor their functions have yet been described in detail. CREBSer^108/111^ and CREBSer^114/117^ can be phosphorylated by CK1B [68], CREBSer^121^ by the ATM kinase/ATR kinase [61] and Ser^129^ by GSK3beta [69]. In contrast, CREBSer^133^ could be phosphorylated by various kinases, such as CaMK4, MAPKSPK2, MSK1, p90RSK or PKD1 [63]. It is noteworthy that phosphorylation is not always sufficient to stimulate CREB-dependent transcription suggesting the existence of additional modifications coordinating CREB activity [2, 70]. The identification of an alternative factor, which can bind CREB independent of its phosphorylation status led to the hypothesis that CREB phosphorylation is not essential for all its physiologic activities.

#### Ubiquitination

Proteasomal degradation represents the primary mechanism of controlled proteolysis and is necessary to maintain cellular function and viability [71, 72]. Targeted proteasomal degradation is also important in the regulation of the expression levels of a number of transcription factors, such as NF-κB and HIF-1 [73]. In addition, CREB can also be targeted for ubiquitination and therefore subsequently degraded by the proteasome. This is important for the quality control of CREB expression and might reflect a mechanism of its fine tuning in response to different stimuli. Recently, a cross talk between different PTMs of CREB has been shown linking hyperphosphorylation of CREB with ubiquitination and its proteasomal degradation [74].

#### Acetylation

CREB protein has been shown to be acetylated by CBP/p300, which in turn affects its transcriptional activity by promoting its DNA-binding capability thereby enhancing its transactivation activities [75, 76]. Furthermore, acetylation of CREB has been linked to its increased protein stability. Although mutations in p300 have been frequently found in human tumors [77, 78], it has not yet been analyzed whether defects in CBP/p300 have a direct effect on the expression level and function of CREB.

#### Glycosylation

Different studies suggested that N-acetylglucosamine (O-GlcNAc) glycosylation can regulate the activity of transcription factors and other proteins in the nucleus [79]. CREB has been shown to be modified by O-GlcNAc at Serin 40, which impairs basal and activation-induced CREB-mediated transcriptional activities [80], thereby modulating important cellular functions. Glycosylation can function as a constant repressor of CREB, which controls its basal expression pattern along with the levels of CREB-regulated genes, such as e. g. Wnt2 and c-fos [81], but this mechanism has not yet been linked to tumorigenesis.

#### SUMOylation

The small ubiquitin-like modifier (SUMO) modification (SUMOylation) is an important mechanism in post-transcriptional control. In most cases SUMOylation suppress the activity of targeted transcriptional activators by altering their sub-compartmentalization and/or protein interaction properties [82, 83]. The short isoform of CREB has been shown to be SUMOylated by the SUMO E3 ligase protein inhibitor of activated STAT1 [55]. Recently, SUMOylation of AKT has been shown to regulate substrate SUMOylation specificity including the targeting of CREB [84].

CREB can also be modified by SUMOylation in response to hypoxia. Overexpression of SUMO1 stabilizes CREB and enhances CREB-dependent gene reporter activity in hypoxia [60]. Lysine residues K304 and K285 of CREB are SUMO1 acceptors demonstrating that SUMOylation represents an important PTM of CREB. The CREB SUMOylation is dependent on its phosphorylation, but lasts longer. The distinct of phosphorylation status and SUMOylation kinetics suggests that CREB phosphorylation is responsible for signal transduction during early responses, while CREB SUMOylation sustains long-term processes [55]. This might also lead to changes within the cellular metabolism by affecting the expression of e.g. mitochondrial genes. Therefore, analysis of the SUMOylation status of CREB in tumor cells is suggested.

### Subcellular localization of CREB

Next to the temporal control by post-translational modifications (PTMs) the CREB activity might be also controlled by its subcellular localization. Although primarily localized in the nuclear compartment, CREB can also be found in the cytoplasm as well as in mitochondria [85]. The importance of the compartment-specific translocation of CREB is in particular demonstrated by monitoring CREB expression in tumors of distinct origin as well as in tumor cells cultured under various conditions mimicking the tumor microenvironment, such as hypoxia and altered pH [86, 87]. Furthermore, PTMs have also been linked to the subcellular localization of CREB. This alters CREB function as shown for mitoCREB [88, 89].

## CREB DOWNSTREAM TARGETS AND THEIR CELLULAR FUNCTIONS IN CANCER

**Figure 3:**
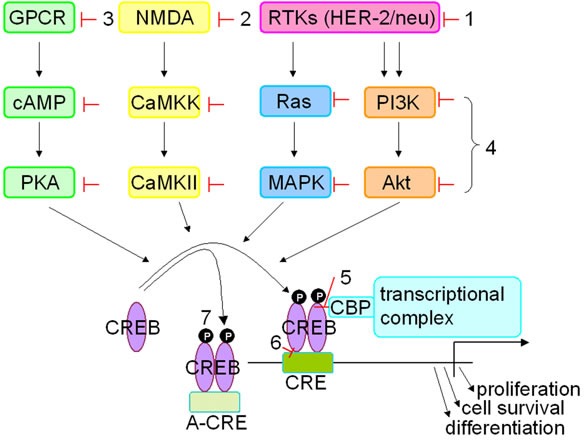
Strategies inhibiting CREB expression Different approaches were used to inhibit/silence CREB expression and/or activity *in vitro* and *in vivo*. These include (i) upstream inhibitors of CREB blocking different receptor tyrosine kinases, like HER-2/neu and EGF-R, with e.g. trastuzumab or lapatinib, (ii) inhibition of the ion transporter (NMDA) by treatment with ketamine, (iii) inactivation of G-protein coupled receptors with beta blockers, (iv) activity of kinases or substrates using various signal transduction inhibitors, (v) inhibition of the interaction between CREB and the co-activators CBP/p300 with KG-501, (vi) influencing binding of CREB at the CRE site by treatment with surfenhydrate and (vii) blocking the interaction with the gene promoter using an artificial CRE element.

The transcriptional activator CREB enhances the expression of many target genes, which are involved in various cell functions including metabolism, cell cycle, survival and DNA repair suggesting that CREB is of critical importance for the growth, survival, migration, as well as for viral responses (Figure 3; [90–92]). This raised the question how CREB acts under pathophysiological circumstances e.g. initiation and progression of tumors and viral infection. The effects of CREB, in particular distinct cellular processes, depend on the proper activation of a specific gene expression program. A distinct set of genes is regulated by CREB under different circumstances as demonstrated by chromatin immunoprecipitation and DNA microarray techniques [32]. The CREB-mediated gene transcription is critical in maintaining a homeostatic cellular environment under pathological conditions. Indeed, there exist a large number of putative CREB target genes, which include genes that are involved in signal transduction, cellular structure, differentiation, cell proliferation as well as metabolism. All target genes exhibit the presence of one or more CRE consensus sequences in their promoter regions. Despite these characteristics the CREB target genes only share a few other similarities reflecting the highly variable activities of CREB under different conditions. The functional diversity of CREB target genes has also intriguing mechanistic implications and their activity may not coordinately be turned on when CREB is phosphorylated or turned off when CREB is de-phosphorylated.

## TARGETING CREB IN TUMOR CELLS

Based on its central role in the development, maintenance as well as progression of tumors CREB has been suggested as an excellent target structure for the treatment of cancers. This is further underlined by the expression analysis of the early inducible cAMP repressor (ICER; inducible cyclic AMP early repressor), an inhibitor of CREB [93]. ICER is downregulated in bone marrow cells of patients with acute myeloid leukemia (AML) leading to an altered CREB expression level. An advantage of CREB as a target is its regulation by different signal transduction pathways, known to be involved in tumor development. So far, different strategies have been developed to inhibit CREB function in tumor cells (Figure 3). These include the use of dominant-negative CREB mutants (KCREB), which could inhibit the transcription of CREB by heterodimerization of KCREB with wild type CREB. Overexpression of KCREB in metastatic tumor cells leads to a reduced potential of metastasis formation both *in vitro* and *in vivo* [94]. Recently, an inhibitor of CREB created by the fusion of the dominant negative inhibitor A-CREB with a photoactive yellow protein was designed controlling CREB function [95]. Thus the link of CREB with optogenetic domains enables the analysis of spatiotemporal control of CREB and its therapeutic use.

Furthermore, a number of CRE „decoy” oligonucleotides have been established, which not only efficiently inhibit CREB gene transcription, but also tumor growth [96]. Using RNA interference CREB expression was silenced, which was associated with altered growth properties and cell viability. In tumor cells the shRNA-mediated inhibition of CREB caused a reduced tumor cell proliferation and migration anchorage-independent growth, suppression of cell cycle arrest and induction of apoptosis accompanied by a reduced *in vivo* tumor growth and enhanced tumor immunogenicity [48, 97].

In addition, an advantage of CREB as a target is its regulation by different signal transduction pathways, which have been shown to be involved in the tumor development. Since these „proof of concept“ studies demonstrated a therapeutic effect of CREB inhibition, alternative strategies using small molecules have been developed to inhibit the CREB-mediated gene transcription. These include the development of different kinase inhibitors, which inhibit the phosphorylation and thus the activation of CREB as well as of chemical inhibitors, which were able to inhibit the interaction between CREB-CRE or CREB-CBP [98–102]. The inhibitor KG-501 is able to reversibly and dose-dependently inhibit the interaction between the KID domain of CREB and the KIX domain of CBP. This inhibition was already obvious at micromolar concentrations without inhibiting the general transcription machinery. Another option is the use of miRs to inhibit CREB expression and activity. Indeed, miRs directly blocking CREB activity and thus the neoplastic phenotype of tumor cells have been recently identified [30], but so far their implementation *in vivo* has not yet been established.

## CONCLUSIONS

It is noteworthy that the disruption of CREB activity has severe consequences and is lethal in mice [103]. The use of sophisticated genetic models might be suitable tools for increasing the knowledge of CREB in survival and maintenance of the cellular fate as well as in its role in many diseases including cancer. The identification of the molecular mechanisms involved in CREB expression and regulation will lead to strategies to inhibit persistent CREB activity in tumors thereby reverting the CREB-induced transformation processes. Thus, CREB might not only serve as a prognostic marker, but also as a therapeutic target for cancers associated with increased activity of signal transduction pathways.

## Abbreviations

AML: acute myeloid leukemia
bZIP: basic leucine zipper
cAMP: cyclic AMP
CBP: CREB-binding protein
ChiP: chromatin immunoprecipitation
CRE: cAMP response element
CREB: cAMP response element binding protein
CREM: FGF fibroblast growth factor
GF: growth factor
ICER: inducible cAMP early repressor
i.e.: immediate early
KCREB: dominant-negative CREB
KID: kinase-inducible domain
KIX: KID interacting domain
MAPK: mitogen-activated protein kinase
MDSC: myeloid-derived suppressor cells
miR: microRNA
NSCLC: non-small cell lung carcinoma
O-Glc NAc: N-acetylglucosamine
pCREB: phosphorylated CREB PI3K phosphatidylinositol 3-kinases PKA protein kinase A
PTM: posttranslational modification
RTK: receptor tyrosine kinase
sh: short hair pin
SUMO: small ubiquitin-like modifier
TF: transcription factor TGF transforming growth factor
TTP: tristetraprolin
UTR: untranslated region.
